# Identification of Novel Imatinib-Resistant Genes in Gastrointestinal Stromal Tumors

**DOI:** 10.3389/fgene.2022.878145

**Published:** 2022-05-13

**Authors:** Lei Cao, Kunming Zheng, Yanhong Liu, Peng Song, Chuntao Wang, Hongzhi Wang, Nan Wang, Shiwu Zhang, Yongjie Zhao

**Affiliations:** ^1^ Department of General Surgery, Tianjin Union Medical Center, Tianjin, China; ^2^ Tianjin Key Laboratory of General Surgery in Construction, Tianjin Union Medical Center, Tianjin, China

**Keywords:** GIST, gastrointestinal stromal tumor, imatinib, DEG (differentially expressed gene) analysis, resistance, gene chip

## Abstract

Gastrointestinal stromal tumors (GISTs) are common ICC precursor sarcomas, which are considered to be a potential malignant mesenchymal tumor driven by specific KIT or PDGFRA signals in the gastrointestinal tract. The standard treatment for GIST without metastasis is surgical resection. GIST with metastasis is usually treated with tyrosine kinase inhibitors (TKIs) only but cannot be cured. The TKI imatinib is the main drug of GIST drug therapy. In adjuvant therapy, the duration of imatinib adjuvant therapy is 3 years. It has been proved that imatinib can improve the overall survival time (OS). However, many GIST patients develop drug resistance due to the long-term use of imatinib. We were forced to look for new strategies to treat GIST. The purpose of the current academic work is to study the drug-resistant genes of imatinib and their potential mechanisms. A total of 897 differentially expressed genes (DEGs) were found between imatinib-sensitive cell line GIST882 and imatinib-resistant cell line GIST430 by RNA sequencing (RNA-seq). After analyzing the DEGs, 10 top genes were selected (NDN, FABP4, COL4A1, COLEC11, MEG3, EPHA3, EDN3, LMO3, RGS4, and CRISP2). These genes were analyzed by RT-PCR, and it was confirmed that the expression trend of FABP4, COL4A1, and RGS4 in different imatinib-resistant cell lines was in accord with the GEO database. It is suggested that these genes may play a potential role in the clinical diagnosis and treatment of imatinib resistance in GIST.

## Introduction

Gastrointestinal stromal tumors (GISTs) are sarcomas mainly derived from the precursor of interstitial cells (ICCs). It is the most common of all sarcomas (Blay, Kang, Nishida, & von Mehren, 2021). GISTs are heterogeneous tumors, including various molecular entities with usually mutually exclusive mutations of activated oncogenes, mainly KIT or platelet-derived growth factor-alpha (PDGFRA) mutations (Heinrich et al., 2008; Gastrointestinal Stromal Tumor Meta-Analysis, 2010). Bleeding, pain, and obstruction are common clinical symptoms of GIST. GISTs are rare tumors, with an incidence of ∼1.2 per 10^5^ individuals (Nilsson et al., 2005). Most GISTs occur in the stomach (60% Mel 65%), followed by the small intestine (20% Mel 25%), while GISTs in the rectum (3–5%), colon (1–2%), and other sites (8–10%) are rare (Casali et al., 2018; Joensuu et al., 2020; von Mehren et al., 2014). In the epidemiological survey of GISTs, the median age is a broad range, estimated to be 60–65 years.

GIST is not classified as benign or malignant but is stratified according to its malignant clinical risk: very low, low, intermediate, or high. Mietinnn et al. demonstrated that the metastatic risk of GIST increases with tumor size, but not with mitotic count (Miettinen, Lasota, & Sobin, 2005). At present, surgical resection is still the main method for the treatment of GIST. GIST with metastasis is usually treated only with tyrosine kinase inhibitors and cannot be cured. Therefore, an early diagnosis is the only way to improve its prognosis. GISTs are resistant to standard cytotoxic therapy for other sarcomas. However, tyrosine kinase inhibitors (TKIs) targeting KIT and/or PDGFRA have significantly improved survival rates. In the context of advanced disease, TKI treatment has significantly increased the median survival time in the past 20 years, from 18 months to more than 5 years (Casali et al., 2018; von Mehren et al., 2014). The majority of these patients benefit from imatinib treatment; however, a large proportion of patients develop imatinib resistance within 2 years. Although some prognostic biomarkers have been exploited, the imatinib resistance of GIST remains weak due to its difficulty in early detection (Daar, 2012).

Therefore, more reliable resistant biomarkers should be explored as a target for improving the treatment effect and better understanding the underlying mechanism (Demetri et al., 2006). Gene chip, which was used for more than 10 years, can quickly detect differentially expressed genes and was proved to be a reliable technique that could make huge data produced and stored in public databases (Zheng et al., 2021). Therefore, a large number of valuable clues could be explored for new research on the basis of these data. Furthermore, many bioinformatics studies on GIST have been produced in recent years (Zhang et al., 2021), which proved that the integrated bioinformatics methods could help us to further study and better explore the underlying mechanisms.

In this study, first, we have chosen GSE89673 from Gene Expression Omnibus (GEO) (Kelly, Gutierrez Sainz, & Chi, 2021). Second, Gene Ontology (GO) and Kyoto Encyclopedia of Genes and Genomes (KEGG) enrichment analyses of the DEGs were performed using the “clusterProfiler” R package. The top ten upregulated genes (NDN, FABP4, COL4A1, COLEC11, MEG3, EPHA3, EDN3, LMO3, RGS4, and CRISP2) were selected for subsequent analysis. Third, we verified these genes and identified three prominent differential expression genes by PCR between the imatinib-sensitive cell line GIST882 and imatinib-resistant cell line GIST430 (London & Gallo, 2020). In conclusion, the bioinformatics analysis of our study provides some additional useful biomarkers, which could be an effective target for GIST patients (W.-K. Huang et al., 2020).

## Materials and Methods

### Identification of Differential Expression Genes

The data of imatinib-sensitive cell lines GIST882 and imatinib-resistant cell lines were downloaded from GSE89673. The data were divided into two groups: imatinib-sensitive cell line group and imatinib-resistant cell line group, and the differential expression of genes between the two groups was analyzed. R language software (R.4.1.2) and R package (“limma”) were used to analyze data sets and filter out DEG (W. K. Huang et al., 2020). “adj.P.Val <0.05” “|logFC|≥2” were taken as the standard. The volcano figure and heatmap were created by “ggplot2” package.

### Enrichment Analysis of DEGs

Gene Ontology (GO) and Kyoto Encyclopedia of Genes and Genomes (KEGG) enrichment analyses of the DEGs were performed using the “clusterProfiler” R package (Heinrich et al., 2020).

### PPI Network Construction and Module Analysis

Search Tool for the Retrieval of Interacting Genes (STRING; http://string.embl.de/) is a powerful online tool for building PPI networks. It can build DEG PPI networks based on known and predicted PPI and then analyze functional interactions between proteins. Based on the online tool STRING, the PPI of DEG is constructed, and the confidence score is ≥0.7. Then, the PPI network is visualized by Cytoscape software (version 3.5.1).

### Quantitative Real-Time PCR (qPCR)

Total RNAs of the samples were isolated using the Absolutely RNA Microprep kit (Agilent Technologies, Santa Clara, CA, United States). Total cDNA was synthesized by the High Capacity cDNA Reverse Transcription Kit (Thermo Fisher Scientific, Waltham, MA, United States). The qPCR was performed by using SYBR Green qPCR mix (Invitrogen, Carlsbad, CA, United States) on a light cycler instrument (Bio-Rad Laboratories, Hercules, CA, United States) (Gelderblom et al., 2020). The primer sequences are listed in Table 1.

**TABLE 1:** Primer sequences used for real-time PCR assay.

Gene	Forward primer (5–3′)	Forward primer (5–3′)
GAPDH	GAA​GGT​GAA​GGT​CGG​AGT​CAA​C	TGG​AAG​ATG​GTG​ATG​GGA​TTT​C
FABP4	ACT​GGG​CCA​GGA​ATT​TGA​CG	CTC​GTG​GAA​GTG​ACG​CCT​T
COL4A1	GGG​ATG​CTG​TTG​AAA​GGT​GAA	GGT​GGT​CCG​GTA​AAT​CCT​GG
RGS4	ACA​TCG​GCT​AGG​TTT​CCT​GC	GTT​GTG​GGA​AGA​ATT​GTG​TTC​AC

## Results

### Identification of Differential Expression Genes

**FIGURE 1:**
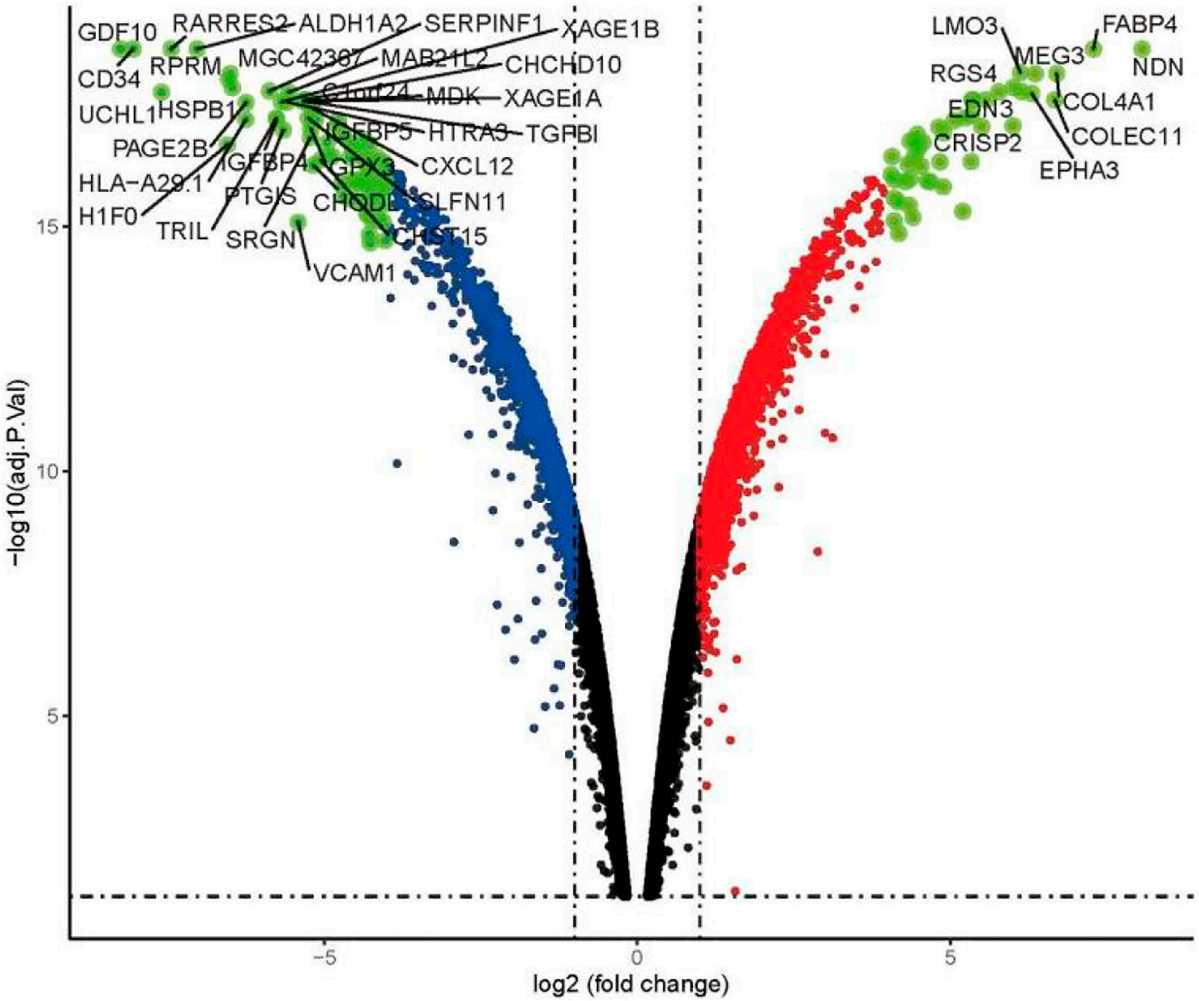
Volcano map of differential expression analysis in GIST882 and GIST430 data sets.

**FIGURE 2:**
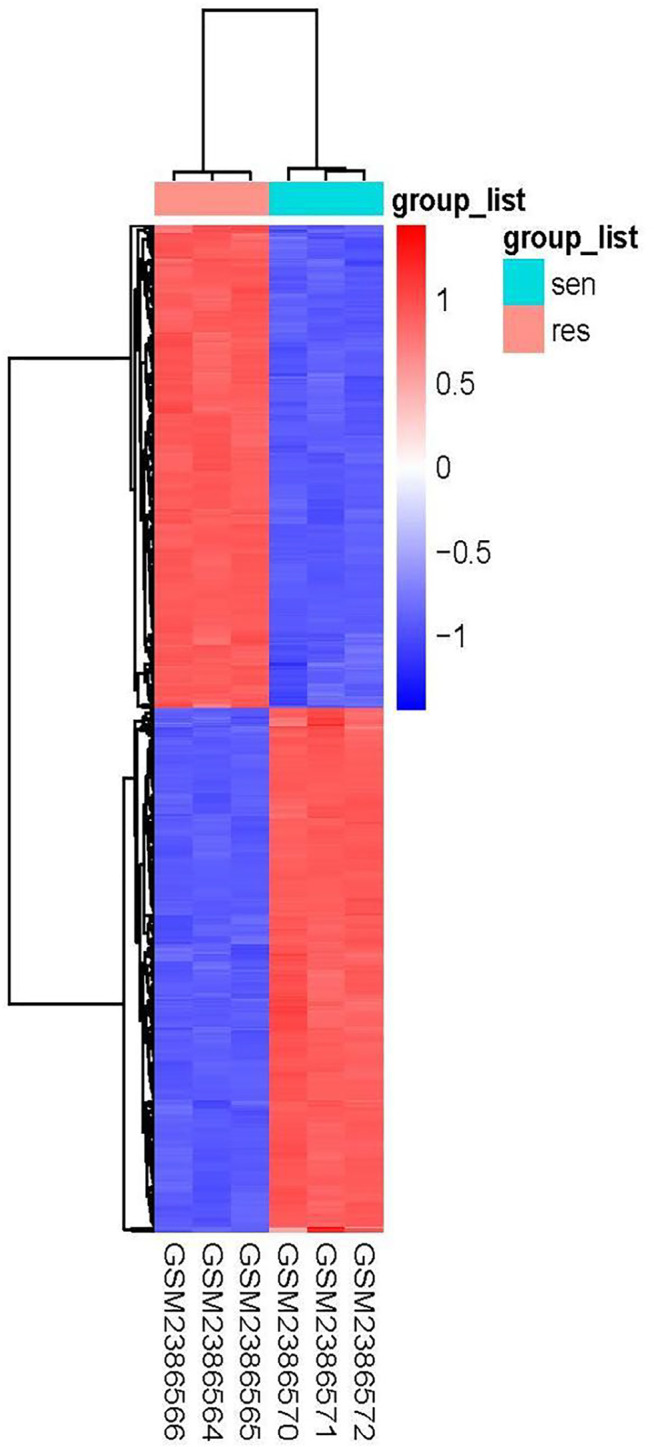
Heatmap of differentially expressed genes in GIST882 and GIST430 data sets.

Using the condition of adj.P.Val <0.05, |logFC|≥2, a total of 897 genes were found to be differentially expressed between the imatinib-sensitive cell line GIST882 and imatinib-resistant cell line GIST430. Of these, 431 genes were upregulated and 466 were downregulated. Differentially Expressed Genes (DEGs) in the two groups are represented in Figure 1. The RNA expression levels of these genes are represented by the heatmap shown in Figure 2.

### Enrichment Analysis of DEGs

**FIGURE 3:**
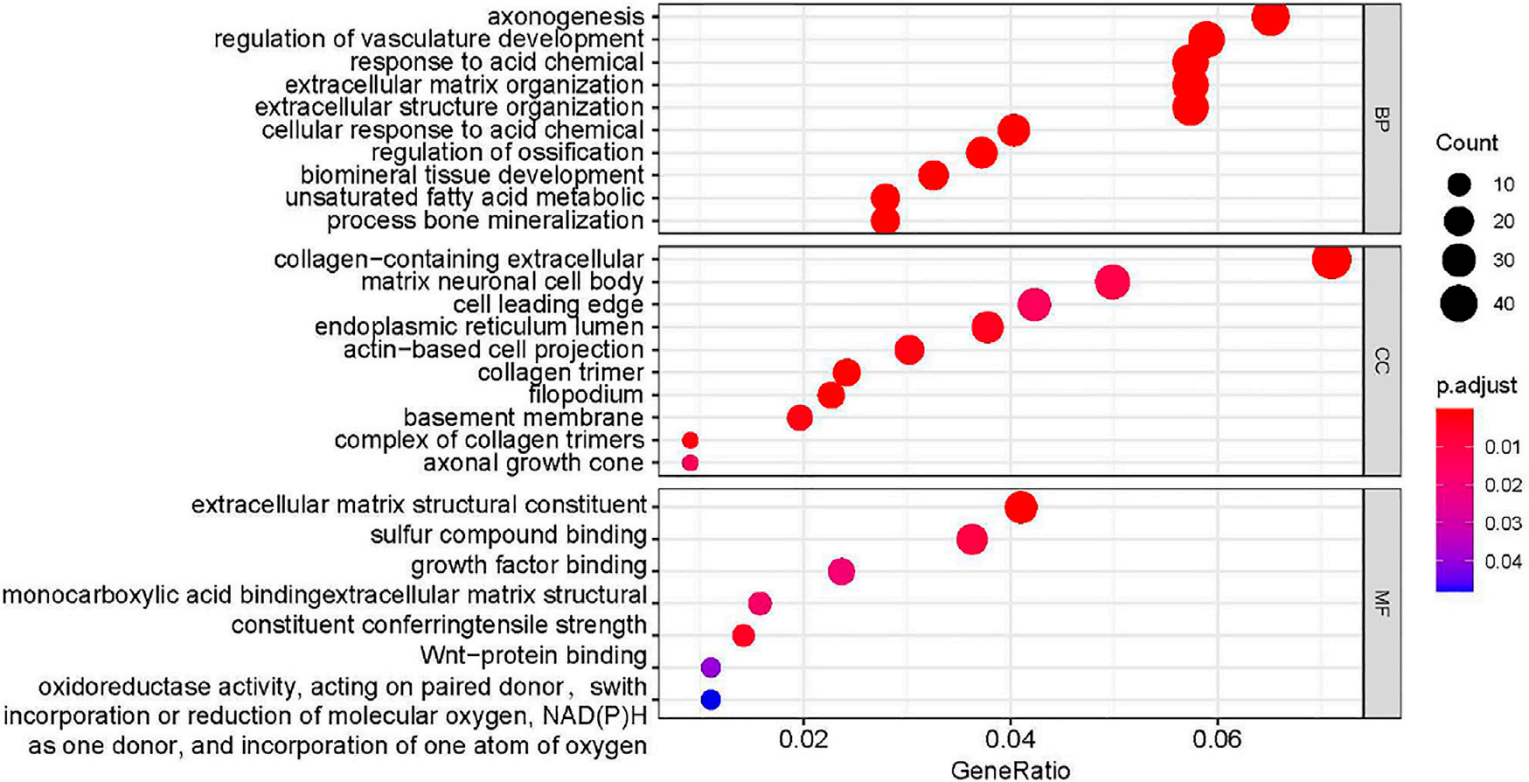
Analysis results of functional enrichment.

**FIGURE 4:**
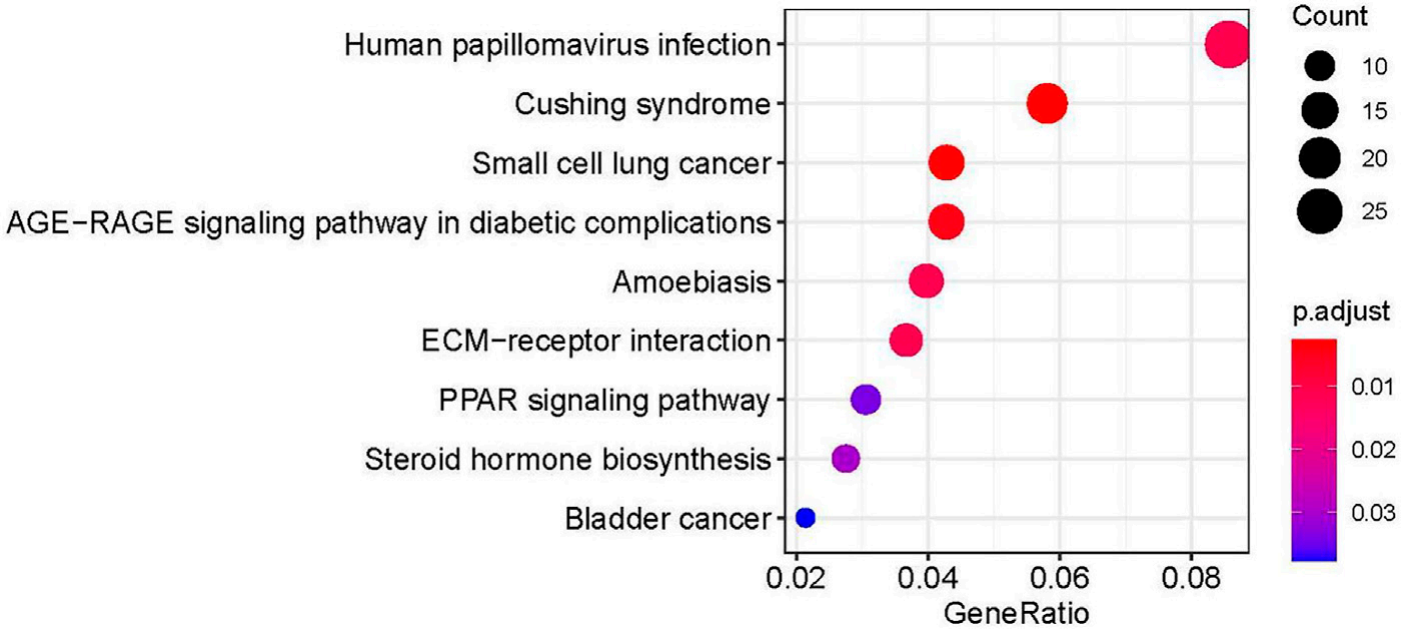
Enrichment analysis by KEGG of DEGS.

In order to further explore the role of DEGs in imatinib-resistant cell lines, GO and KEGG enrichment analyses were undertaken on obtained DEGs. The DEGs were mainly involved in the positive regulation of axonogenesis, regulation of vasculature development, response to acid chemical, collagen-containing extracellular matrix, neuronal cell body, cell leading edge, extracellular matrix structural constituent, sulfur compound binding, and growth factor binding in GO analysis (Figure 3). Moreover, KEGG pathway analysis suggested that DEGs were mainly involved in human papillomavirus infection (Blay et al., 2020), Cushing syndrome, small-cell lung cancer, AGE−RAGE signaling pathway in diabetic complications, amebiasis, ECM−receptor interaction, PPAR signaling pathway, steroid hormone biosynthesis, and bladder cancer (Figure 4).

### Experimental Validations of DEGs

**FIGURE 5:**
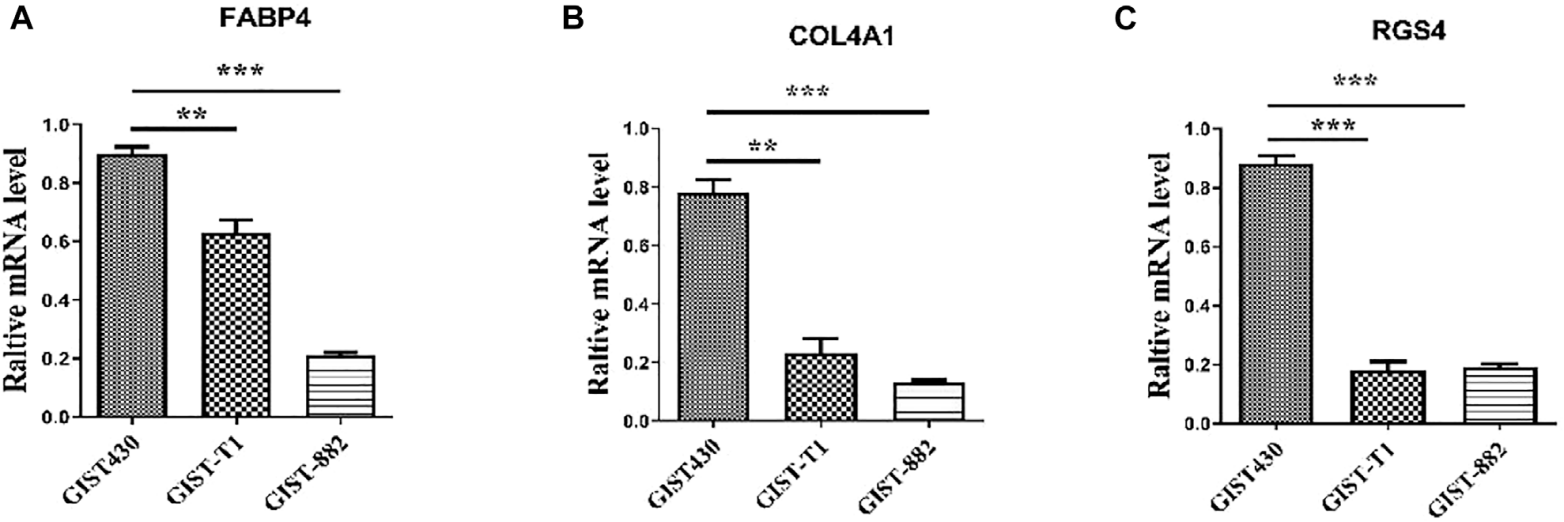
Expression levels of FABP4, COL4A1, and RGS4 in different GIST cell lines determined using quantitative real-time polymerase chain reaction. “**” means *p*-value < 0.01, “***” means *p*-value < 0.001.

The top ten upregulated genes (NDN, FABP4, COL4A1, COLEC11, MEG3, EPHA3, EDN3, LMO3, RGS4, and CRISP2) were selected for subsequent analysis (Table 2). After screening candidate genes, qPCR was used to verify these candidate genes. After three kinds of GIST cells were treated with imatinib, the following figure showed that the expression of FABP4, COL4A1, and RGS4 in imatinib-sensitive lines GIST882 and GIST-T1 decreased significantly compared with GIST430 cell lines. This is consistent with the database results (Figure 5).

**TABLE 2:** Top ten upregulated genes of DEGs.

Gene symbol logFC AveExpr P.Value adj.P.Va
NDN 8.0670 11.0970 3.35E-23 2.37E-19
FABP4 7.2881 10.7183 1.83E-23 2.37E-19
COL4A1 6.7008 10.2284 2.42E-22 7.60E-19
COLEC11 6.6720 10.5963 2.01E-21 2.60E-18
MEG3 6.3592 10.2336 2.31E-22 7.60E-19
EPHA3 6.3025 9.8446 1.16E-21 1.92E-18
EDN3 6.1380 9.6867 8.42E-22 1.76E-18
LMO3 6.1217 9.6960 1.60E-22 7.20E-19
RGS4 6.0082 10.2738 5.82E-22 1.45E-18
CRISP2 6.0010 9.8171 1.19E-20 8.87E-18
Abbreviations: FC, fold change; AveExpr, average expression quantity; adj.P.Va, adjusted P value

### Protein Product Co-Expression Network Analysis

**FIGURE 6:**
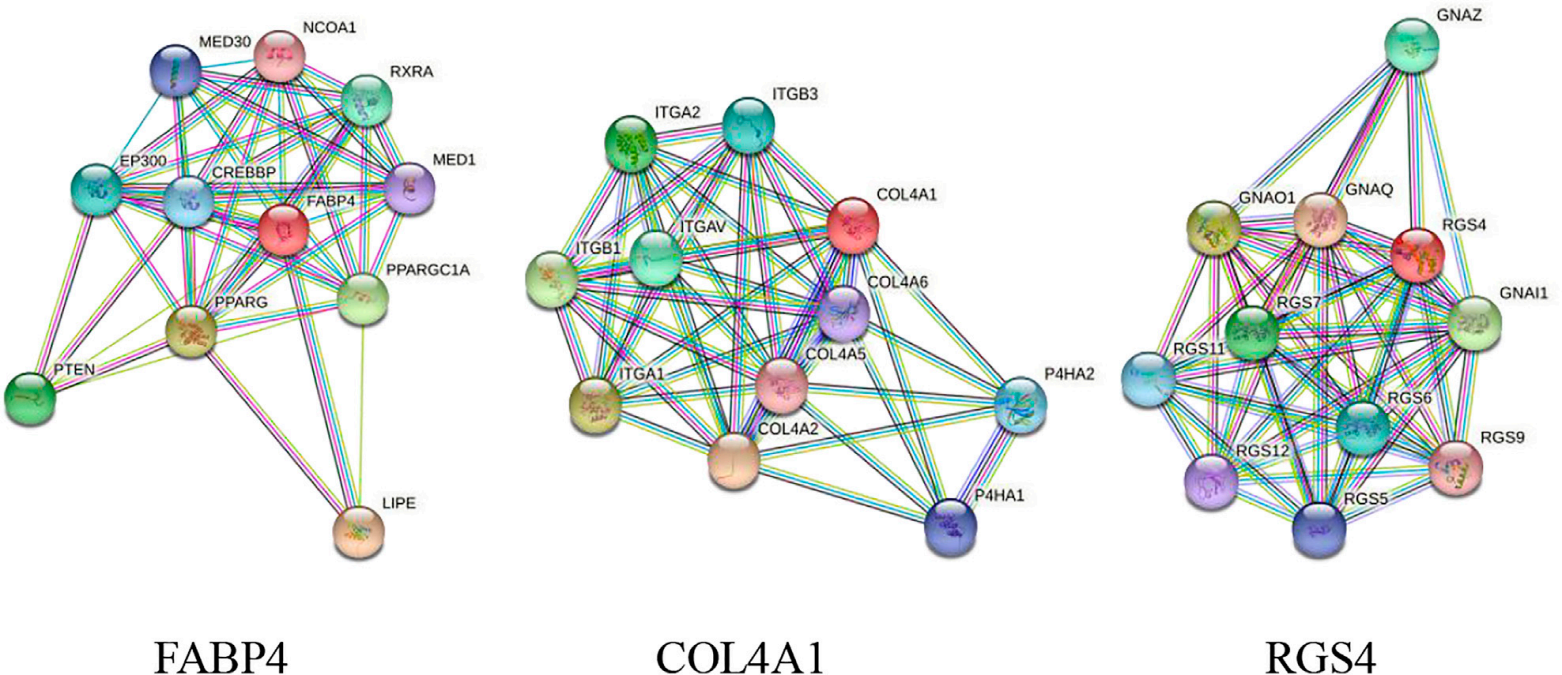
PPI network of DEGs obtained from the STRING database. The protein network was calculated based on the neighborhood score with higher confidence (confidence score >0.99).

The FABP4, COL4A1, and RGS4 genes were studied for possible interactions with each other using the STRING database. It was predicted that these DEGs would have significant interactions. The PPI network contained 33 numbers of nodes (each node indicates proteins), and the edges present interactions. The FABP4 network showed the enriched co-expressed genes (PPI enrichment, *p* < 0.05) functionally associated with mediator of RNA polymerase II transcription subunits 1 and 30 (MED1 and MED30), nuclear receptor coactivator 1 (NCOA1), retinoic acid receptor RXR-alpha (RXRA), CREB-binding protein (CREBBP), peroxisome proliferator-activated receptor (PPARG), phosphatase and Tensin homolog (PTEN), and hormone-sensitive lipase (LIPE). COL4A1 is directly connected to the integrin (ITG) family source genes, COL4A (collagen alpha) family source genes, and prolyl 4-hydroxylase subunit alpha (P4HA) family source genes. Similarly, it has been observed that RGS4 interacts with important target proteins such as regulator of G-protein signaling (RGS) family source genes and guanine nucleotide-binding protein G (GNA) family source genes (Figure 6).

## Discussion

To identify more useful biomarkers of resistance to imatinib in GIST, this study used bioinformatics methods on the basis of GSE89673 datasets (Toulmonde et al., 2019). We analyzed mRNA expression profile chip data GSE89673, which compared the mRNA expression changes of the drug-resistant cell line GIST430 with the sensitive cell line GIST882. The mRNA expression changes of the sensitive cell line GIST882 were further analyzed. There were 897 differential genes, of which 431 were upregulated and 466 were downregulated. GO and KEGG enrichment analyses were undertaken on obtained DEGs. The DEGs were mainly involved in the positive regulation of axonogenesis, regulation of vasculature development, response to acid chemical, collagen-containing extracellular matrix, neuronal cell body, cell leading edge, extracellular matrix structural constituent, sulfur compound binding, and growth factor binding in GO analysis. Moreover, KEGG pathway analysis suggested that DEGs were mainly involved in the human papillomavirus infection, Cushing syndrome, small-cell lung cancer, AGE−RAGE signaling pathway in diabetic complications, amebiasis, ECM−receptor interaction, PPAR signaling pathway, steroid hormone biosynthesis, and bladder cancer.

Fatty acid-binding protein 4 (FABP4), also known as adipocyte FABP, is mainly expressed in adipocytes and macrophages. Elevated levels of circulating FABP4 are associated with obesity, insulin resistance, diabetes, hypertension, cardiac dysfunction, atherosclerosis, and cardiovascular events (Y. Zhang et al., 2021). It has been shown that the knockdown of FABP4 leads to increased 5-levels of hydroxymethylcytosine in DNA, downregulation of key genes associated with ovarian cancer metastasis, and reduced survival of replication to cancer cells (Furuhashi, Saitoh, Shimamoto, & Miura, 2014). More studies have shown that high FABP4 expression in advanced serous ovarian cancer cells reduces the rate of metastatic tumor growth in mice. Thus, the small-molecule inhibitor (BMS309403) of FABP4 not only significantly reduced tumor load in syngeneic *in situ* mouse models but also increased cancer cell sensitivity to carboplatin both *in vitro* and *in vivo*. Lipid desaturation of SCD1 in cancer cells and the lipid transport of FABP4 produced by tumor endothelial cells (TECs) promote the survival of cancer cells and the resistance to iron death in the TME. The blockade of FABP4 and SCD1 activity in tumors inhibited these processes and significantly reduced tumor recurrence (Wan, Guo, Zhu, & Qu, 2020). FABP4 is highly expressed in cancer tissues and is associated with TNM stage, differentiation, and lymph node metastasis in colorectal cancer studies (Y. Zhang et al., 2021).

COL4A1 is the major anti-angiogenic gene induced by p53 in human adenocarcinoma cells, and p53 directly activates the transcription of the COL4A1 gene by binding to its 26-kbp enhancer region downstream of the 3’ ending (Mukherjee et al., 2020). Some studies have analyzed 206 surgical pathology specimens from breast cancer and adjacent tissues using immunohistochemical staining with antibodies specific to COL4A1 and evaluated the correlation between clinical results and the IHC score of COL4A1 (Wang et al., 2020). The correlation between COL4A1 expression and long-term OS and RFS in breast cancer patients was further investigated by Kaplan–Meier analysis. The results showed that COL4A1 is associated with breast cancer prognosis (Plaisier & Ronco, 1993). Through a comprehensive screening of the expression profiles of collagen genes, COL4A1 was the most differentially expressed collagen gene in HCC. Proliferation and metastasis of HCC cells were promoted by FAK-Src signaling after upregulation of COL4A1 (Y. Zhang et al., 2021). Recent studies show that COL4A1 expression is upregulated by the transcription factor RUNX1 and found that HCC cells with high COL4A1 expression are sensitive to the treatment of FAK or Src inhibitors. It is concluded that COL4A1 may be a potential target for the diagnosis and treatment of HCC (Wang et al., 2020).

Regulators of G protein signaling 4 (RGS4) are negative regulators of G protein signaling, and elevated RGS4 levels have been reported to be associated with a variety of human diseases, including cancer (Xue, Wang, Meng, Jiao, & Dang, 2017). RGS4 is an important regulator of melanocyte apoptosis, and the rate of apoptosis is significantly reduced at low RGS4 expression levels. RGS4 induces inactivation of the PI3K/AKT pathway, resulting in reduced E2F1 and cyclin D1 expression with the effect of limiting cell proliferation and invasion (Guda, Velpula, Asuthkar, Cain, & Tsung, 2020). Recent studies have shown that the different proteins of the RGS family are all involved in tumor development. For example, overexpression of RGS1 inhibited CXCL12-mediated human plasmacytoma cell migration, and epigenetic inhibition of RGS2 has been associated with prostate cancer progression and overexpression of RGS5 on lung cancer cells (He et al., 2019). The overexpression of RGS17 promotes the propagation of lung tumor cells through the circulating AMP-PKA-CREB pathway (Cheng et al., 2016). Therefore, there is a consensus suggesting that RGS proteins can be used as potential candidates for tumor diagnosis and treatment (Xiao & Gao, 2019).

Numerous studies have proved that FABP4, COL4A1, and RGS4 were related to various types of cancer progression; however, we searched GIST on PubMed with no reports on our screened differential genes and GIST. Therefore, the data in our study could provide useful information and direction for future study on GIST (Vincenzi et al., 2018).

## Conclusion

In sum, our bioinformatics analysis study identified DEGs between the imatinib-sensitive cell line GIST882 and imatinib-resistant cell line GIST430 on the basis of a microarray dataset. The results showed that FABP4, COL4A1, and RGS4 could play key roles in the imatinib resistance of GIST (Serrano et al., 2019). However, these predictions should be verified by a series of experiments in the future (Duan et al., 2019). Anyway, these data may provide some useful information and direction into the potential biomarkers and biological mechanisms of GIST (Hu et al., 2019).

## Data Availability

The datasets presented in this study can be found in online repositories. The names of the repository/repositories and accession number(s) can be found in the article.
